# Calcitriol decreases HIV-1 transfer *in vitro* from monocyte-derived dendritic cells to CD4 + T cells, and downregulates the expression of DC-SIGN and SIGLEC-1

**DOI:** 10.1371/journal.pone.0269932

**Published:** 2022-07-08

**Authors:** Natalia Alvarez, Sandra M. Gonzalez, Juan C. Hernandez, Maria T. Rugeles, Wbeimar Aguilar-Jimenez

**Affiliations:** 1 Facultad de Medicina, Grupo Inmunovirología, Universidad de Antioquia UdeA, Medellín, Colombia; 2 Department of Medical Microbiology and Infectious Diseases, University of Manitoba, Winnipeg, MB, Canada; 3 Facultad de Medicina, Infettare, Universidad Cooperativa de Colombia, Medellín, Colombia; Tulane National Primate Research Center, UNITED STATES

## Abstract

Dendritic cells (DCs) promote HIV-1 transmission by acting as Trojan horses, capturing viral particles, facilitating the infection of CD4+ T-cells. Vitamin D (VitD) has shown to decrease T cell activation, reducing susceptibility to HIV-1 infection of CD4+ T-cells in vitro; however, if VitD decreases viral transfer from DCs to CD4+ T-cells is unknown. In this study, we co-cultured HIV-1-pulsed immature and LPS mature monocytes-derived DCs (iDCs and LmDCs, respectively), differentiated in presence or absence of calcitriol (VitD active form), with PHA-activated autologous CD4+ T-cells from 16 healthy donors. In co-cultures of iDCs and LmDCs treated with calcitriol, there was a significant decrease in frequency of infected CD4+ T-cells, evaluated by flow cytometry. However, p24 levels evaluated by ELISA were not significantly reduced in culture supernatants. Moreover, calcitriol-treated iDCs exhibited decreased expression of genes involved in HIV-1 transfer compared to the control. Both, calcitriol-treated iDCs and LmDCs exhibit a similar gene expression profile, probably related to a transcriptional balance achieved after long treatment with calcitriol. Since calcitriol-differentiated DCs express on their surface a lower amount of DC-SIGN and SIGLEC-1 molecules, widely associated with HIV-1 transfer, suggesting that this mechanism contributes to a lower transfer of viral particles by the DCs.

## 1 Introduction

HIV-1 infection remains a global public health issue that has caused approximately 32 million deaths since the beginning of the HIV pandemic [1]. The failure to find a sterilizing cure has made the combined antiretroviral therapy (cART) the only efficient alternative that is currently available to treat infected individuals. Furthermore, the search for immunomodulatory compounds that may reduce the risk of acquiring the infection or delay AIDS progression is one of the field’s priorities.

Calcitriol arises as a good candidate since, beyond its role in mineral metabolism, it has anti-inflammatory [2,3] and antimicrobial functions [4–6]. Remarkably, higher VitD levels in plasma and expression of its receptor (VDR) in blood cells and mucosa were found in HIV-exposed seronegative individuals (HESN) compared to seropositive and HIV-unexposed healthy subjects, suggesting that this vitamin is likely associated with natural resistance to HIV-1 infection [7]. Indeed, it has been observed that *in vitro* and *ex vivo* treatment with VitD reduces the frequency of HIV-infected CD4+ T cells [8–10]. Such reduction appears to be explained by reduced immune activation and induction of antiviral gene expression [9,10]; however, further exploration confirming these findings is required.

In addition to its modulatory effects on CD4+ T cells [9,10], VitD has been shown to decrease the activation and maturation profile of dendritic cells (DCs) [11–13]; therefore, it could reduce the ability of these cells to transfer HIV-1 viral particles to CD4+ T cells, contributing to reducing the viral spread that usually occurs during the initial stages of infection [14].

The viral transfer may occur by two mechanisms: cis- and trans-infection [15,16]. Cis-infection follows a productive infection in DCs, in which new viral progeny infects the CD4+ T cells through the classical CD4 receptor [17,18]. Trans-infection occurs when DCs transfer viral particles without being infected by keeping them attached to its cell membrane or trapping them in non-lysosomal compartments until the viral synapse with CD4+ T cells occurs [18]. In the trans-infection process, receptors such as DC-SIGN and SIGLEC-1 are involved, which are mainly expressed in immature DCs (iDCs) and mature (mDCs) DCs, respectively [15]. Cellular processes and several proteins related to membrane trafficking, actin formation and stabilization, and vesicle and microtubule formation are also critical to mediate trans-infection [19]. Expression of dynamin 2 (DNM2) and tetraspanin 7 (TSPAN7) in DCs favors maintaining viral particles on the surface of DCs, whereas DCs deficient in DNM2 and TSPAN7 redistributed viral particles in micropinosomes, exhibiting reduced trans-infection ability [19].

Based on this previous evidence, we evaluated the effect of the active form of the VitD, calcitriol, on HIV-1 transfer from DCs to CD4+ T cells, using an *in vitro* model of acute infection. Since DCs exhibited a reduced susceptibility to infection with X4-tropic viruses [20], the trans-infection path might be favored in this model. Monocytes-derived dendritic cells (hereafter referred to as DCs) were cultured in the presence or absence of calcitriol, pulsed with HIV-1 and co-cultured with autologous CD4+ T cells. The frequency of HIV-1-infected CD4+ T after co-culture was evaluated. Moreover, the relative expression of genes associated with vesicle trafficking and dendrites formation, potentially modulated by calcitriol, such as *CD63*, *VAMP3*, *DNM2*, *MYO5*, and *TSPAN7* were also evaluated. Finally, the expression of receptors DC-SIGN and SIGLEC-1, key molecules mediating viral attachment, were also explored.

## 2. Materials and methods

### 2.1. Study population

Peripheral blood mononuclear cells (PBMCs) were isolated through a blood density gradient using the Histopaque reagent (Sigma-Aldrich) from 16 healthy donors from the blood bank of the "Escuela de Microbiologia, UdeA", Medellin-Colombia, all of them tested negative for HIV 1/2, HCV, HBV, HTLV 1/2, Chagas, and Syphilis. They were aged between 18 and 50 years where 63% of donors were men. The exclusion criteria were individuals with immunosuppressive or anticoagulant drug treatments, individuals who reported any chronic disease, and pregnant women. The study was performed according to the Helsinki declaration (1975, revised in 2000), and was approved by the Bioethics Board of the Instituto de Investigaciones Médicas, Universidad de Antioquia (ACT-008-2016). Informed consent was obtained from all subjects involved in the study.

### 2.2. Preparation of monocyte-derived dendritic cells

Monocytes were obtained from PBMCs by CD14-negative selection, using the Miltenyi, Human Pan Monocyte Isolation Kit, according to the manufacturer’s instructions. The cells obtained were cultured for 7 days using RPMI medium (Sigma-Aldrich) supplemented with 10% fetal bovine serum (FBS) (Sigma-Aldrich), 1% antibiotic (Penicillin-Streptomycin) (Sigma-Aldrich), GM-CSF at 75ng/mL and IL-4 at 50 ng/mL (Prepotech). Half of the cells were differentiated in the presence of 5×10^−9^ M of calcitriol (Sigma-Aldrich) (Calcitriol concentrations found in serum after calcitriol supplementation [21,22] and in culture supernatants produced by DCs *ex vivo* [23,24]) and the remaining cells in the presence of 0,01% vol/vol EtOH as vehicle control, obtaining a cell viability greater than 95% (S1 Fig). Half of the culture medium was replenished every two days, maintaining the initial components’ same concentration. After six days of culture, and once the DCs were differentiated [25], half of the cells from each treatment (Calcitriol or EtOH) were treated with or without 5 μg/mL ultrapure lipopolysaccharide (LPS) for 24 hours to obtain LPS mature (LmDCs) and immature (iDCs) DCs, respectively. For practical purposes, the cells treated with LPS are referred to as LmDCs regardless of the profile obtained after differentiation or not with calcitriol.

### 2.3. Isolation and activation of autologous CD4+ T cells

Autologous CD4+ T cells were obtained by CD4-negative selection, using Miltenyi, CD4+ T cell Isolation Kit, according to the supplier’s instructions. The cells obtained were cultured for 48 hours in RPMI medium at 10% FBS, 1% antibiotic, supplemented with 8 μg/mL phytohemagglutinin (PHA), and 50 IU/uL of IL-2 (Sigma-Aldrich) for activation, thus increasing the susceptibility to HIV-1 infection.

### 2.4. HIV-1 transfer assay (co-culture)

LmDCs and iDCs were pulsed for 2 hours with X4-tropic HIV-1 virions (15 ng of p24), obtained from supernatants of the H9-HTLV-IIIB cell line. The mixture of viruses and DCs were washed three times with PBS to remove non-absorbed virions. HIV-loaded DCs were co-cultured with activated autologous CD4+ T cells in a 1:3 proportion (50,000 DCs and 150,000 CD4+ T cells) in RPMI 1640 medium supplemented with 10% FBS and 1% antibiotic. After 72 hours, p24 expression was evaluated by flow cytometry, and p24 levels were measured by ELISA (XpressBio, Frederick, MD). The gating strategy to evaluate the percentage of CD4 T cells infected was summarized in the S2 Fig.

### 2.5. CD4+ T cells infection with free viral particles

As a control of the intrinsic susceptibility of infection of autologous CD4+ T cells by free viral particles, PHA/IL-2 activated CD4+ T cells were pulsed for 2 hours with X4-tropic HIV-1 virions (15 ng of p24), then washed twice with PBS to remove non-absorbed virions. Cells were cultured for 7 days in RPMI 1640 medium supplemented with 10% FBS and 1% antibiotic. Levels of p24 were measured using the ELISA Kit XB-1000 (XpressBio, Frederick, MD), 3- and 7-day post-infection.

### 2.6. Flow cytometry

For each assay, cell viability was tested with efluor 506 staining. Expression of activation and maturation markers in DCs was determined by staining with anti-HLA-DR FITC, anti-CD11c efluor 450, anti-CD80 PE-cyanine5, anti-CD86 PE-cyanine7, anti-CD40 PE, and anti-CD83 APC (all from eBioscience). Also, the expression of DC-SIGN and SIGLEC-1 in DCs was determined using anti-CD209 PerCP-cyanine5.5 and anti-CD169 super bright 600 (eBioscience). The frequency of infected CD4+ T cells was evaluated by detecting intracellular p24 (anti-p24 PE, Beckman-coulter, Brea, CA) (S2 Fig). Fluorescence minus one (FMO) or isotype controls were performed for all markers used. The acquisition was performed on an LSR Fortessa (BD) flow cytometer, and analysis was performed using the FlowJo v.7.6 software.

### 2.7. Real-time PCR

To assess the effects of calcitriol on the expression of genes involved in HIV-1 transfer in DCs, the genes implicated in HIV-1 transference, *CD63*, *VAMP3*, *DNM2*, *TSPAN7*, *MYO5*, *SIGLEC1*, *CD209*, and that according to the database of transcription factor binding profiles JASPAR, have possible VDREs, were selected for gene expression [19,26,27]. Total RNA from cells of each treatment was extracted with the Direct-zol RNA Kit (Zymo Research); following DNAse I treatment (Thermo Fisher Scientific), the RNAs were retrotranscribed using a recombinant Moloney Murine Leukemia Virus retrotranscriptase (Thermo Fisher Scientific). Reverse transcriptase negative controls were performed to rule out contamination with genomic DNA in PCR amplifications. Real-time PCRs were performed using the Maxima SYBR Green qPCR Master Mix 2x (Thermo Fisher Scientific), running melting curves to ensure specific amplification. The results are presented as the relative expression units’ median to the *ACTB* reference gene calculated by the ΔCt method. The RNAs were processed as described above to evaluate the expression of HIV-1 restriction factors *SAMHD1*, *TRIM5* and *APOBEC3G* in autologous CD4+ T cells. The results are presented as the relative expression units’ median to the β-Actin reference gene calculated by the ΔCt method.

### 2.8. Statistical analysis

Data were analyzed on the GraphPad Prism V.7.05 software. The Shapiro-Wilk test tested normality, and pairwise differences between treatments were tested by the paired t-test or Wilcoxon test, depending on the normality fulfillment. Since the number of cells of some of the 16 individuals was not enough to perform all the experiments, sample sizes are specified in the figure legends. Results are presented as mean, and p-value < 0.05 was considered statistically significant.

## 3. Results

### 3.1. Calcitriol decreased maturation and activation of DCs

VitD has been reported to promote an immature profile in DCs with tolerogenic properties [11–13]. We validated the effect of the presence of calcitriol during the differentiation of DCs, in the expression of maturation and activation markers by flow cytometry. The DCs cultured for 7 days and maturated with LPS (LmDCs) or maintained in an immature state (iDC), although with some variability, faithfully reproduce these two DCs phenotypes according to the expression of CD11c, CD86 and HLA-DR (S3 Fig) as previously reported [28,29]. These two phenotypes, iDC and LmDC differ on their expression of the maturation marker CD83 as expected (S3 Fig). Moreover, as predicted, calcitriol treatment reduced the expression of CD83, CD40, CD80, and CD86, measured by median fluorescence intensity (MFI), in both iDCs (by 67%, p = 0.0081; 67%, p = 0.0355; 70%, p = 0.0023; and 57%, p = 0.022, respectively) and LmDCs (by 75%, p < 0.0001; 64%, p = 0.0625; 46%, p = 0.0012; and 50%, p = 0.0132 respectively) compared to the control (S4 Fig).

Likewise, the presence of the calcitriol treatment during the differentiation process of these cells, also reduced the percentage of cells expressing the markers CD83, CD40, and CD80, but not those expressing CD86 in both, iDCs (by 90%, P = 0.0025; 83%, P = 0.0252; 36%, P = 0.0248; and 48%, P = 0.519 respectively) and LmDCs (by 96%, p = 0.0002; 84%, p = 0.0476; 17%, p = 0.394; and 42%, p = 0.3125 respectively) (S4 Fig). Additionally, no significant differences were observed in the expression of these markers when comparing iDCs and LmDCs treated with calcitriol (P >0.062 for each marker, S4C vs S4D and S4E vs S4F Fig), suggesting that this treatment generates a stable immature-like phenotype even after an inflammatory stimulus.

### 3.2. The percentage of infected CD4+ T cells is lower when co-cultured with HIV-1 pulsed calcitriol-treated DCs

**Fig 1:**
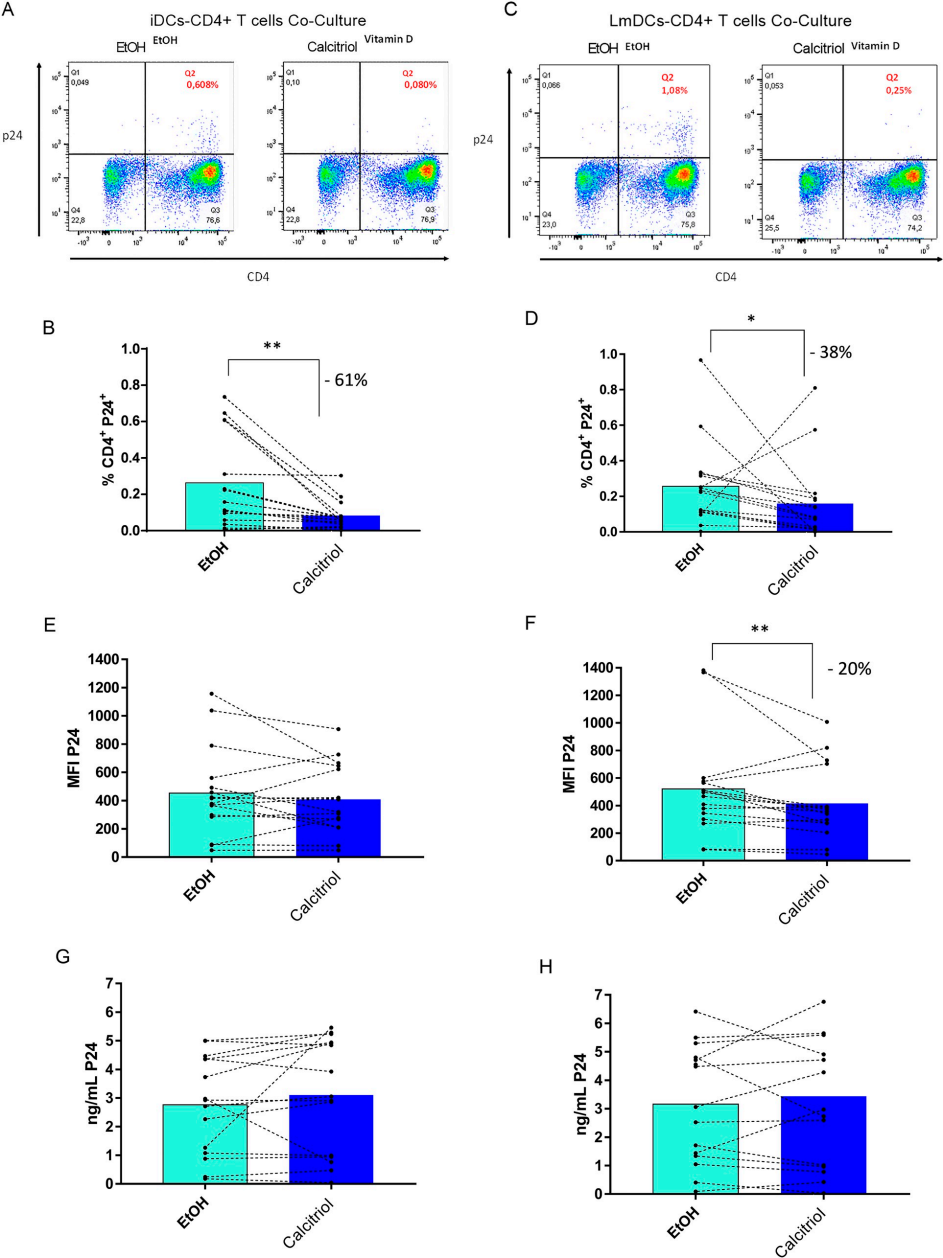
Percentage of infected CD4+ T cell is reduced when co-cultured with HIV-1 pulsed calcitriol-treated DCs. iDCs (left panel) and LmDCs (right panel) were pulsed with X4 tropic virions (H9-HTLV-IIIB) and co-cultured with CD4+ T cells. After 72 h, the viral protein p4 levels were evaluated by flow cytometry (n = 16) and ELISA (n = 15). The doplots corresponding to gate strategy to select the infected CD4 populations are represented in the figures A for iDCs and C for LmDCs. The % of P24+ CD4+ T cells (B and D), P24 MFI (E and F), and p24 concentration in ng/mL (G and H) in co-cultures with iDCs and LmDCs were represented in bar charts, where white bars correspond to co-cultures made with DCs treated with EtOH and blue bars to those treated with calcitriol. The statistical analysis was performed using Ratio paired tests except for the % of P24+ CD4+ T cells and P24 concentration in co-cultures with LmDCs where a Wilcoxon test was used due to non-normal distribution of differences. Bars represent the mean, *P < 0.05, **P < 0.01, ***P < 0.001, and ****P < 0.0001, and percent (%) decrease are depicted.

**Fig 2:**
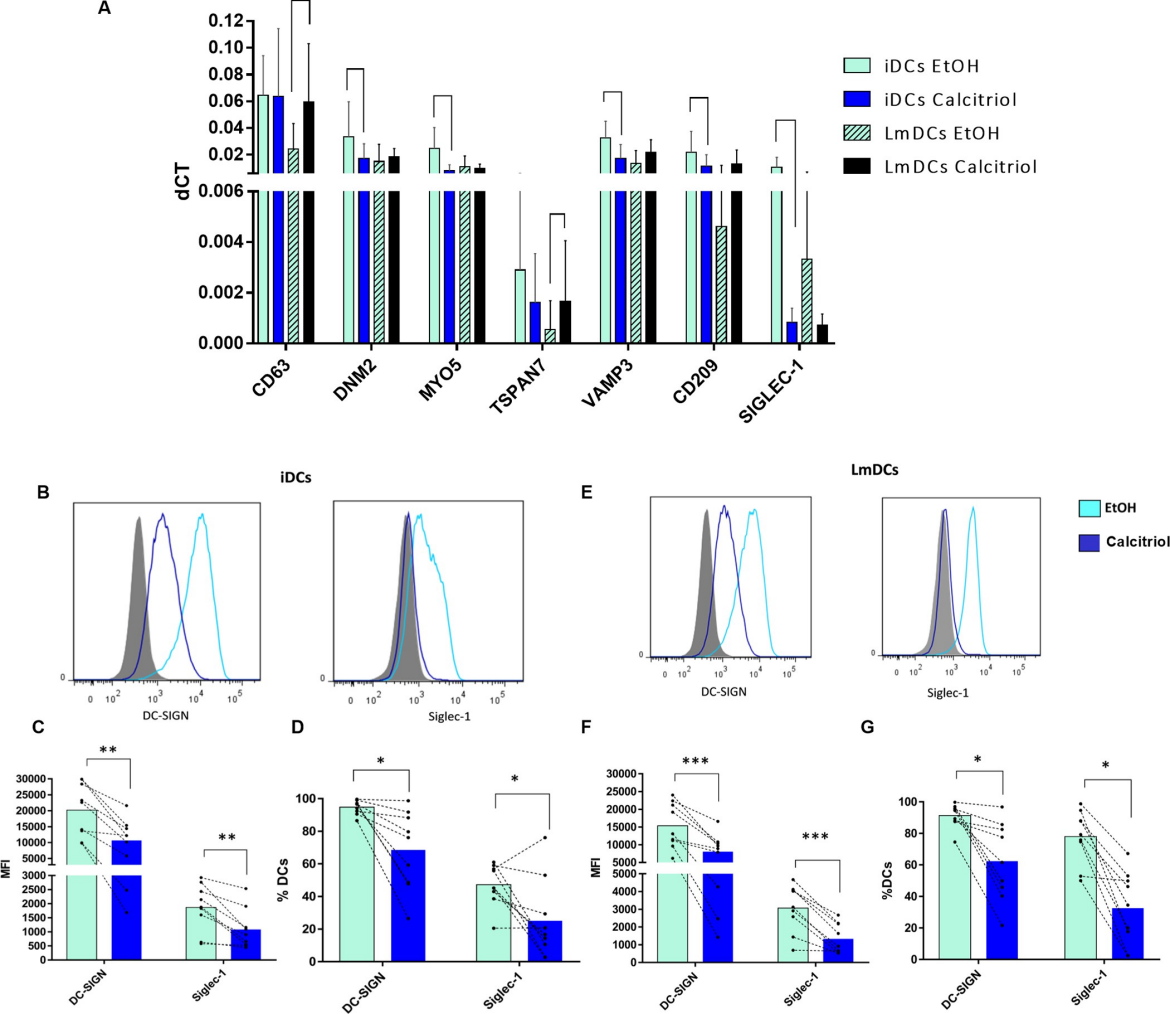
Relative expression of genes that participate in routes related to trans-infection (2A). The gene expression levels were measured by qPCR using ACTB as a reference gene, showing the results in bar graphics representing the median of the relative expression in 7 individuals. Solid bars correspond to iDCs and dashed bars to LmDCs, where white bars correspond to EtOH- treated cells and blue bars to calcitriol-treated cells in each one. The statistical analysis was performed using a Ratio paired test; however, due to the non-normal distribution of data, a Wilcoxon test was used for the *DNM2*, *TSPAN7* and *VAMP3* analyzes LmDCs. Expression of DC-SIGN and SIGLEC-1 in both calcitriol- and EtOH-treated iDCs and LmDCs. (B and E) Representative overlay histograms comparing the expression of DC-SIGN and SIGLEC-1 on FMO control cells (gray fill), calcitriol treated cells (blue lines), and EtOH-treated cells (cyan lines) in both iDCs (B) and LmDCs (E). In the bar graph, cyan bars correspond to cells treated with EtOH and blue bars to cells treated with calcitriol, where the MFI and % in iDCs (C and D) and LmDCs (F and G) of DC-SIGN and SIGLEC-1 are represented as the mean of 8 individuals. *P < 0.05, **P < 0.01, ***P < 0.001, and ****P < 0.0001.

Following the observed reduction in the expression of maturation and activation markers in DCs by calcitriol, we evaluated whether the treatment with this hormone is related to changes in the HIV-1 transfer from DCs to CD4+ T cells, considering that iDCs have been associated with a lower ability of viral transfer [30,31]. For this purpose, we used an *in vitro* model, which consisted of HIV-1 pulsed calcitriol-treated iDCs and LmDCs that were co-cultured with autologous PHA-activated CD4+ T cells. As described in a previous report from our team [10], no difference was found in viral transfer when comparing iDCs and LmDCs (1.69% P24+ CD4+ T cells in iDCs vs 2.39% P24+ CD4+ T cells in LmDCs, p = 0.1053). However, calcitriol treatment in iDCs and LmDCs significantly reduced the frequency of P24+ CD4+ T cells co-cultured with them by 61% (p = 0.0049. Fig 1A and 1B) and by 38% (p = 0.0389. Figs 1C and 2D), respectively, compared to the control counterparts after 3 days of co-culture.

Although the MFI of p24 did not change for CD4+ T cells co-cultivated with calcitriol-treated iDCs (4.5%, p = 0.6979. Fig 1E), a significant decrease by 20% was observed in CD4+ T cells co-cultivated with calcitriol-treated LmDCs compared to the control (p = 0.0077. Fig 1F). Nonetheless, p24 levels in supernatants detected by ELISA, were similar in co-cultures with calcitriol- and EtOH-treated iDCs and LmDCs (Fig 1G and 1H).

### 3.3. The levels of p24 post-infection did not correlate with the transcriptional expression of cellular restriction factors

The similar levels of p24 in co-culture supernatants, despite the reduced frequency of infected T cells after calcitriol treatment, could be explained by differences in the intrinsic susceptibility of CD4+ T cells. To explore this hypothesis, we performed infection assays in CD4+ T cells using free viral particles and evaluated the transcriptional expression of the viral restriction factors *SAMHD1*, *TRIM5*, and *APOBEC3G*. No correlations were found between the concentration of p24 (ng/mL) on day 3 or day 7 (S5 Fig) post-infection, and the expression of the three restriction factors, suggesting that the CD4+ T cells did not exhibit a natural resistance against HIV-1 infection.

### 3.4. While calcitriol-treated iDCs and LmDCs express similar levels of genes related to trans-infection, the protein expression of DC-SIGN and SIGLEC-1 was reduced with the calcitriol treatment

HIV-1 transfer from DCs comprises cell processes such as dendrites formation and vesicle trafficking [19], so we analyzed the expression of genes in these pathways by qPCR in DCs (S1 Table). We found a differential expression of these genes between EtOH-treated iDCs and LmDCs (Fig 2A. Solid cyan vs. cyan dashed bars). Moreover, in calcitriol-treated iDCs, a significant reduction by 48% in *DNM2* (p = 0.0202), 68% in *MYO5* (p = 0.0109), 54% *VAMP3* (p = 0.0455), 53% *CD209* (p = 0.318), and 92% *SIGLEC1* (p = 0.001) was observed compared to EtOH-treated iDCs (Fig 2A).

In calcitriol-treated LmDCs, the expression of some genes was increased compared to the EtOH-treated LmDCs, being significant for *CD63* and *TSPAN7* (183% p = 0.0399, 160% p = 0.0313 respectively. Fig 2A dashed bars); reflecting a return to expression levels that occurs in an immature state in these cells. However, calcitriol-treated iDCs and LmDCs maintain gene expression at similar levels (Fig 2A. Dark blue bars vs. Black bars).

On the other hand, since DC-SIGN and SIGLEC-1 (Encoded by the *CD209* and *SIGLEC1* respectively) have been strongly associated with HIV-1 transference [27,32], we further explored the expression of both receptors in calcitriol-treated iDCs and LmDCs by flow cytometry. First, we observed that MFI of DC-SIGN receptor was significantly decreased upon maturation, while the percentage of DC-SIGN-expressing cells did not change between iDCs and LmDCs as previously reported [33]. For its part, both percentage and MFI of SIGLEC-1 increased upon maturation as expected (S6 Fig).

Remarkably, there was a significant reduction in the MFI expression of DC-SIGN and SIGLEC-1 (Fig 2B and 2C) and in the frequency (Fig 2D. DC-SIGN: MFI p = 0.0016, % p = 0.0182. SIGLEC-1: MFI p = 0.0026, %p = 0.0286) for the calcitriol-treated iDCs compared to EtOH-treated iDCs. Similarly, in calcitriol-treated LmDCs, there was a significant decrease in both proteins in MFI (Fig 2E and 2F) and in the percentage (Fig 2G. DC-SIGN: MFI p = 0.0009, %p = 0.0150. SIGLEC-1: MFI p = 0.0007, %p = 0.0102). These results suggest that the expression of DC-SIGN and SIGLEC-1 in DCs can be modulated by calcitriol, likely contributing to reduce viral transfer.

## 4. Discussion

The search for immunomodulatory compounds that reduce the risk of acquiring HIV-1 infection is one of the research priorities in this field. Based on previous evidence showing the anti-HIV-1 effects induced by the VitD on T cells [8–10] as well as its tolerogenic properties on DCs [11], we evaluated the effect of this hormone on HIV-1 transfer from DCs to CD4+ T cells, which is a critical step to establish the infection, using an in vitro model of acute infection.

We confirmed in our *in vitro* model that calcitriol decreases the activation and maturation markers in DCs, in agreement with previous reports [12,13,34,35]. Calcitriol-treated DCs maintained an immature-like profile even in the presence of LPS stimulus.

Notably, in contrast with literature reports, in this study and in a previous one, recently reported by us [36], there were no differences in viral transfer between iDCs and LmDCs. One possible explanation for the discordant results might rely on differences in the tropism of the strain used and the sensitivity of the methods employed for detecting HIV-1 infection. Previous studies reporting higher HIV-1 transfer to CD4+ T cells by mature DCs compared to iDCs used R5 strains and recombinant viruses with reporter genes [14,37], whereas we used an X4-tropic virus. It has been shown that iDCs preferentially transfer R5-tropic viruses through cis-infection, whereas mature DCs use the trans-infection pathway. In contrast, X4-tropic virus are predominantly transferred by trans-infection regardless the maturation state of the DCs [30]. Such difference could be partially explained by a higher susceptibility to infection of DCs by R5-tropic in contrast to X4-tropic viruses [20,38]. Although the use of an R5-tropic virus could have been more suitable to reflect the events occurring *in vivo* during the earlier stages of infection, given that the main goal of this study was to explore the effect of calcitriol regardless infection pathway, mediated by DCs, the use of an X4-tropic virus is useful for this model.

Likewise, the duration of the co-culture assay might also have contributed to this observation. It has been observed that iDCs preferentially (but not exclusively), transfer HIV-1 by cis-infection, a mechanism that is favored after 24 hours of culture. In contrast, trans-infection is primarily used by LmDCs [30]. We maintained both iDCs and LmDCs in co-culture for three days so that we may have missed any differences occurring earlier after infection.

In this study, we observed that when DCs were differentiated in the presence of calcitriol, the percentage of infected p24+ CD4+ T cells was reduced, an effect that was maintained regardless of induction of maturation of DCs by LPS stimulation. This finding contrasts with our previous observations using the inactive form, cholecalciferol to treat DCs after differentiation, which did not affect viral trans-infection [36]. However, we did not observe differences at the p24 levels in supernatants from co-cultures of EtOH- or calcitriol-treated DCs. This could suggest that calcitriol may reduce viral transfer without altering the subsequent viral replication on pre-activated CD4+ T cells. However, it is also possible that other variables could have influenced the results; for instance, the low efficiency or percentage of infected T-cells, or a higher productive infection in DCs, perhaps contributing to the p24 levels detected in the supernatants. In this study, we did not explore the susceptibility of infection of DCs. In addition, the period of the co-cultures and detection of total p24 levels rather than the protein associated with viral particles might have also affected the results. To solve these hypotheses, it is necessary to carry out additional experiments with different co-culture times, and also controlling the levels of infection of DCs.

Although intrinsic susceptibility of autologous CD4+ T cells could also contribute to explain the similar levels of p24 after transference, viral replication on CD4+ T cells seems not to depend on the viral restriction factors *SAMHD1*, *TRIM5*, or *APOBEC3G* since no correlation between their mRNA expression and p24 levels in supernatants of infected lymphocytes were found.

To elucidate the mechanism by which calcitriol-treated DCs decreased the frequency of infected CD4+ T cells, the expression of genes, previously reported influencing viral transfer, was evaluated [19,27,39]. Although all evaluated genes, except *MYO5A*, are related to increased HIV-1 transfer mediated by DCs [19], we observe that EtOH-treated iDCs present a higher relative gene expression than LmDCs. This result could be related to the enhanced ability of iDCs to capture and endocyte antigens, a process that also involves some of the evaluated genes. However, since we did not find differences in transference mediated by iDCs or LmDCs, other mechanisms may be involved in transference in the context of X4-tropic viruses. Furthermore, as we did not evaluate these proteins, we cannot rule out that despite the increased mRNA expression in iDCs, post-translational modifications occurring at the protein level can affect their expression, contributing to explain our results on viral transference. In addition, is critical to note that even with an increase in the relative gene expression in calcitriol-treated LmDCs of *CD63* and *TSPAN7*, genes that favor viral transfer, and a decrease in calcitriol-treated iDCs of *MYO5* that limit viral transfer, the overall result was a decrease in HIV-1 transfer. These results suggest that other mechanisms, unexplored here, are involved in this process.

In addition, we observed that calcitriol-treated iDCs and LmDCs had similar relative expression levels for all genes, showing a transcriptional balance achieved after the hormone treatment during the differentiation process.

Finally, our results suggest that calcitriol can decrease the protein expression of SIGLEC-1 and DC-SIGN, receptors widely related to trans-infection [27,32], suggesting that this is at least one of the possible mechanisms by which calcitriol reduces viral transfer to CD4+ T cells. One possible bias in our results is that the level of maturation in LmDCs was variable among the subjects possibly challenging the generalization of our results. Experiments overexpressing these proteins in the presence of calcitriol treatment are required to confirm their participation on the effects observed by this hormone. Also, to establish if the calcitriol can impact additional routes is necessary to study more genes and proteins associated with related pathways.

This exploratory study serves as a basis for continuing exploring the calcitriol as a potentially safe and cost-effective therapeutic strategy for reducing the risk of acquiring HIV-1 infection. Remarkably, considering that, beyond its anti-inflammatory and tolerogenic potential and its association with the HIV-1 resistance profile exhibited by HIV-1-exposed seronegative individuals (HESNs) [40,41], this hormone might impact key steps associated directly with viral spread. Additionally, VitD could also have beneficial effects on HIV-1 infected individuals since this hormone decreases inflammation, which is one of the main drivers of the deleterious effects of the infection, pointing its likely use as adjunctive therapy to cART.

## Supporting information

S1 Fig Frequency of viable iDCs and LmDCs after treatment with EtOH or calcitriol by flow cytometry. (TIFF)

S2 Fig Gating strategy for selecting infected CD4 T cells. The cells were selected according to size (FCS) and granularity (SSC). Aggregates were excluded and the region for infected CD4 T cells was established according to expression of CD4 and using FMO for the p24 positive region.
(TIFF)

S3 Fig Frequency and MFI of activation and maturation markers expressed on iDCs and LmDCs. Gating strategy for selecting iDC and mDCs according to FCS and SSC, expression of CD11c and HLA-DR, and expression of maturation marker CD83 (A). Histograms of DCs markers CD11c and HLA-DR, activation markers CD80, CD86, CD40 and maturation marker CD83 and respective FMO controls (B). Frequency and MFI of CD83 in iDC and LmDC (C). Frequency of iDC and LmDCs expressing HLA-DR^high^, CD40, CD80 and CD86 (D). Statistical analysis was performed using a Ratio paired test. *P < 0.05, **P < 0.01, ***P < 0.001, and ****P < 0.0001.
(TIFF)

S4 Fig Calcitriol reduces the expression of maturation and activation markers in both iDCs and LmDCs. iDCs (left panel) and LmDCs (right panel) were differentiated in the presence of 5×10^−9^ M of calcitriol or 0.01% vol/vol EtOH (control vehicle). (A and B) Representative overlay histograms comparing the expression of CD83, CD40, CD80, and CD86 markers on unstained EtOH treated cells (grey fill), calcitriol treated cells (dark blued lines), and EtOH treated cells (cyan lines) in both iDCs (A) and LmDCs (B). In the bar graph, cyan bars correspond to cells treated with EtOH and blue bars to cells treated with calcitriol. MFI (C and D) and % (E and F) in iDCs and LmDCs, correspond to the expression of different markers in 4 to 8 individuals. Statistical analysis was performed using a Ratio paired test. Bars represent the mean*P < 0.05, **P < 0.01, ***P < 0.001, and ****P < 0.0001.
(TIFF)

S5 Fig Correlations between the concentrations of p24 (ng/mL) and the relative expression of viral restriction factors in infected CD4+ T cells. The p24 levels were measured by ELISA test from the supernatant of the infected CD4+ T cells cultures (n = 4) at 3 (A) and 7 days (B) pos-infection. The gene expression levels for *SAMHD1* (green circles), *TRIM5* (black square), and *APOBEC3G* (A3G) (blue triangle) were measures by q-PCR using *ACTB* as a reference gene. Correlations were evaluated using the Pearson coefficient rank (r).
(TIFF)

S6 Fig DC-SIGN and SIGLEC-1 expression in both both iDCs and mDCs EtOH treated. About the DC-SIGN receptor, the MFI was decreased significantly upon maturation (p = 0,0084), while the percentage of cells did not change. For the SIGLEC-1 expression, both percentage and MFI increased upon maturation, (p = 0,0026% and p = 0,0003 MFI).
(TIFF)

S1 Table The crude Cycle Threshold data of gene expression. The crude Cycle Threshold data of gene expression is shown. The primer amplification efficiency of actin amplification was also tested by a serial dilution of one cDNA and we found as expected the slope of the standard curve was -3.455 (efficiency close to 94%).
(XLSX)
