# Supplementary figures and images for: Calcitriol decreases HIV-1 transfer in vitro from monocyte-derived dendritic cells to CD4 + T cells, and downregulates the expression of DC-SIGN and SIGLEC-1
Source: PLoS One. 2022 Jul 8;17(7):e0269932. doi: 10.1371/journal.pone.0269932 (PMC9269915; doi:10.1371/journal.pone.0269932)

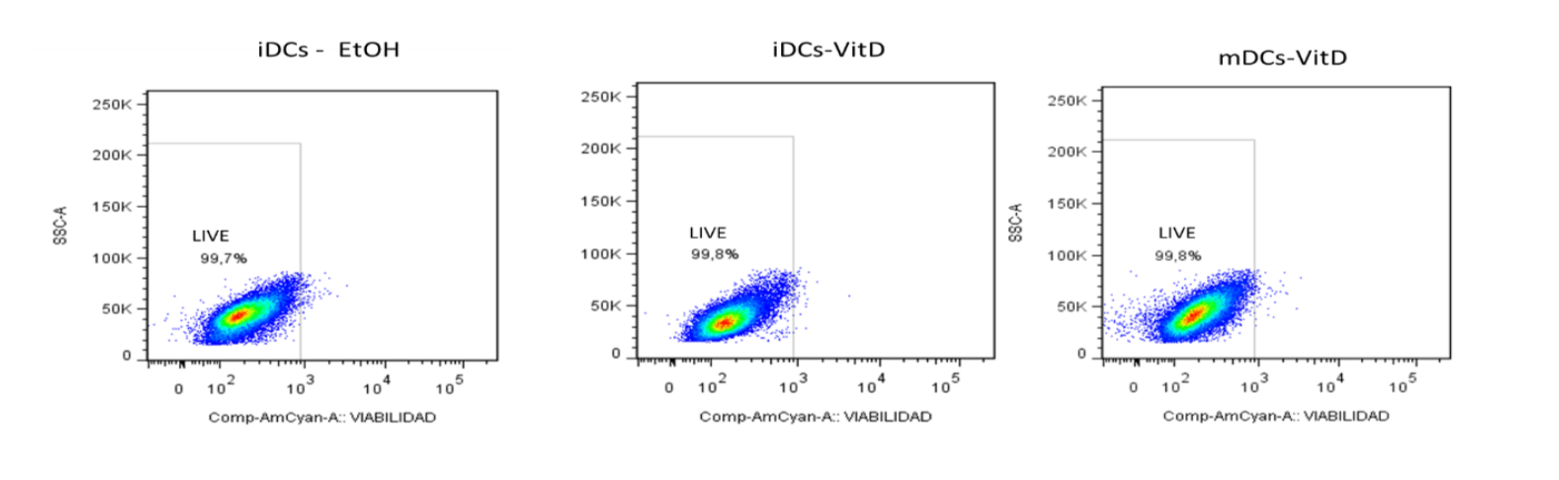

Supplement: S1 Fig — (TIFF) [file pone.0269932.s001.tiff]

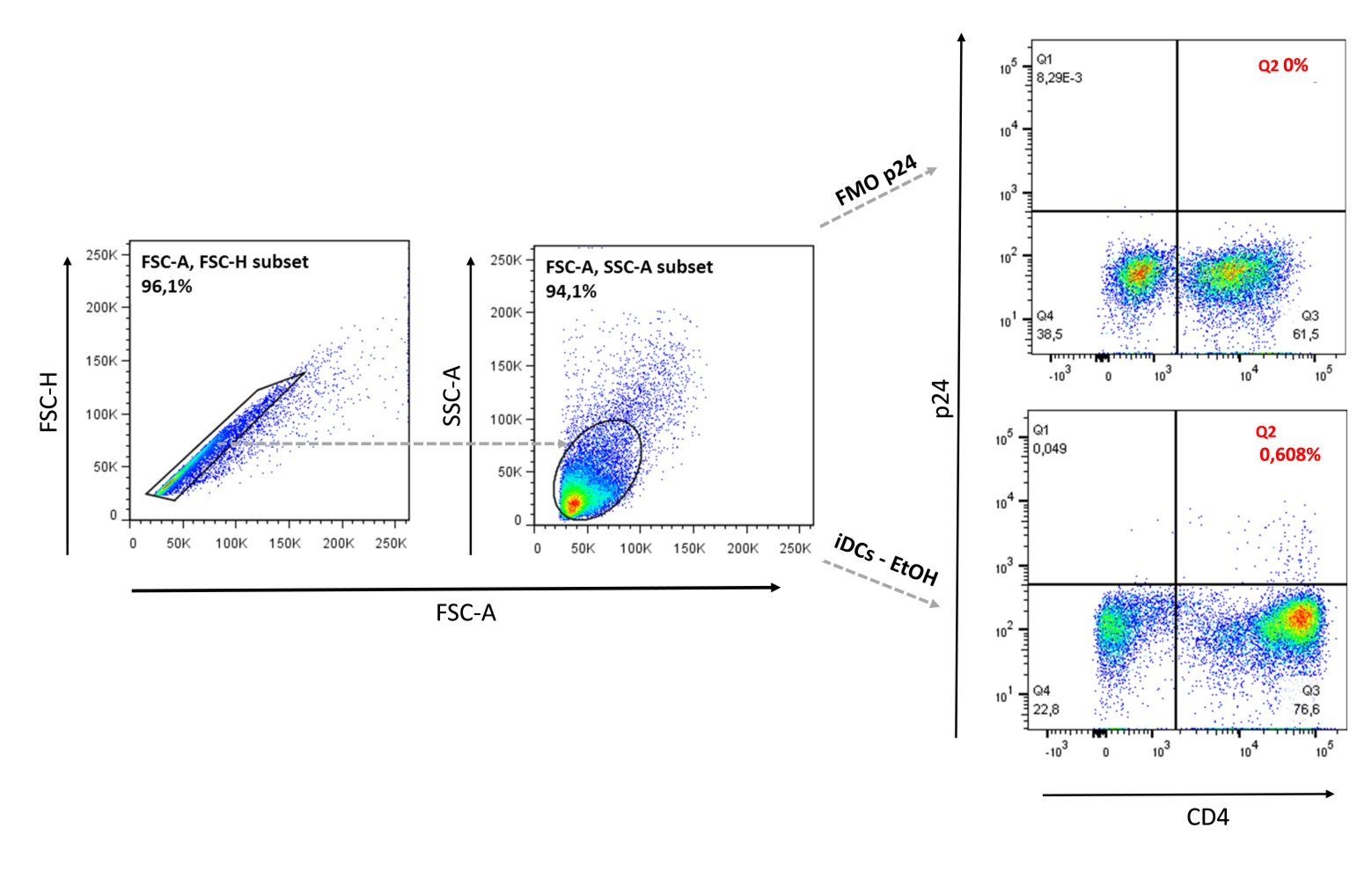

Supplement: S2 Fig — The cells were selected according to size (FCS) and granularity (SSC). Aggregates were excluded and the region for infected CD4 T cells was established according to expression of CD4 and using FMO for the p24 positive region. (TIFF) [file pone.0269932.s002.tiff]

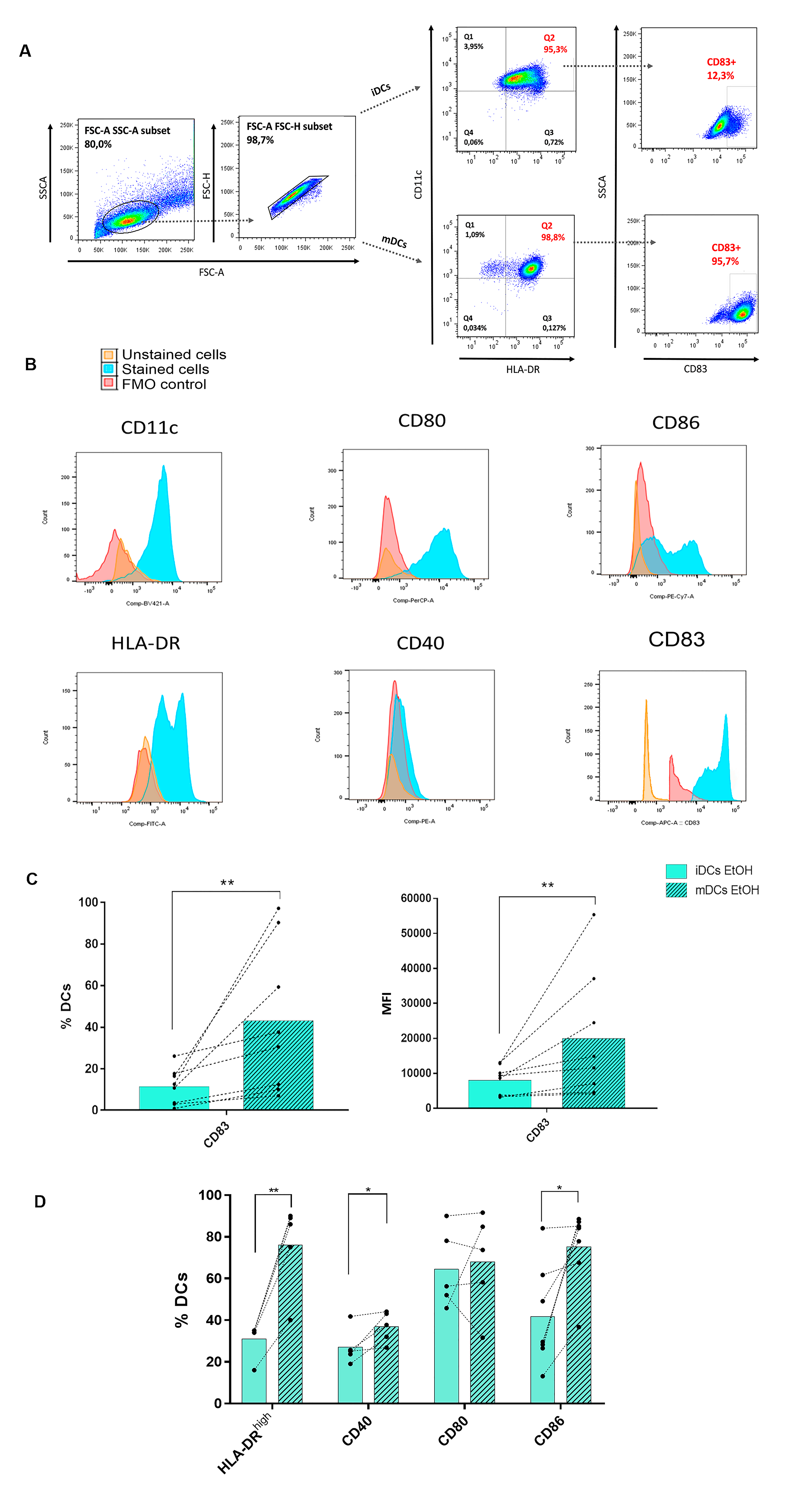

Supplement: S3 Fig — Gating strategy for selecting iDC and mDCs according to FCS and SSC, expression of CD11c and HLA-DR, and expression of maturation marker CD83 (A). Histograms of DCs markers CD11c and HLA-DR, activation markers CD80, CD86, CD40 and maturation marker CD83 and respective FMO controls (B). Frequency and MFI of CD83 in iDC and LmDC (C). Frequency of iDC and LmDCs expressing HLA-DRhigh, CD40, CD80 and CD86 (D). Statistical analysis was performed using a Ratio paired test. *P < 0.05, **P < 0.01, ***P < 0.001, and ****P < 0.0001. (TIFF) [file pone.0269932.s003.tiff]

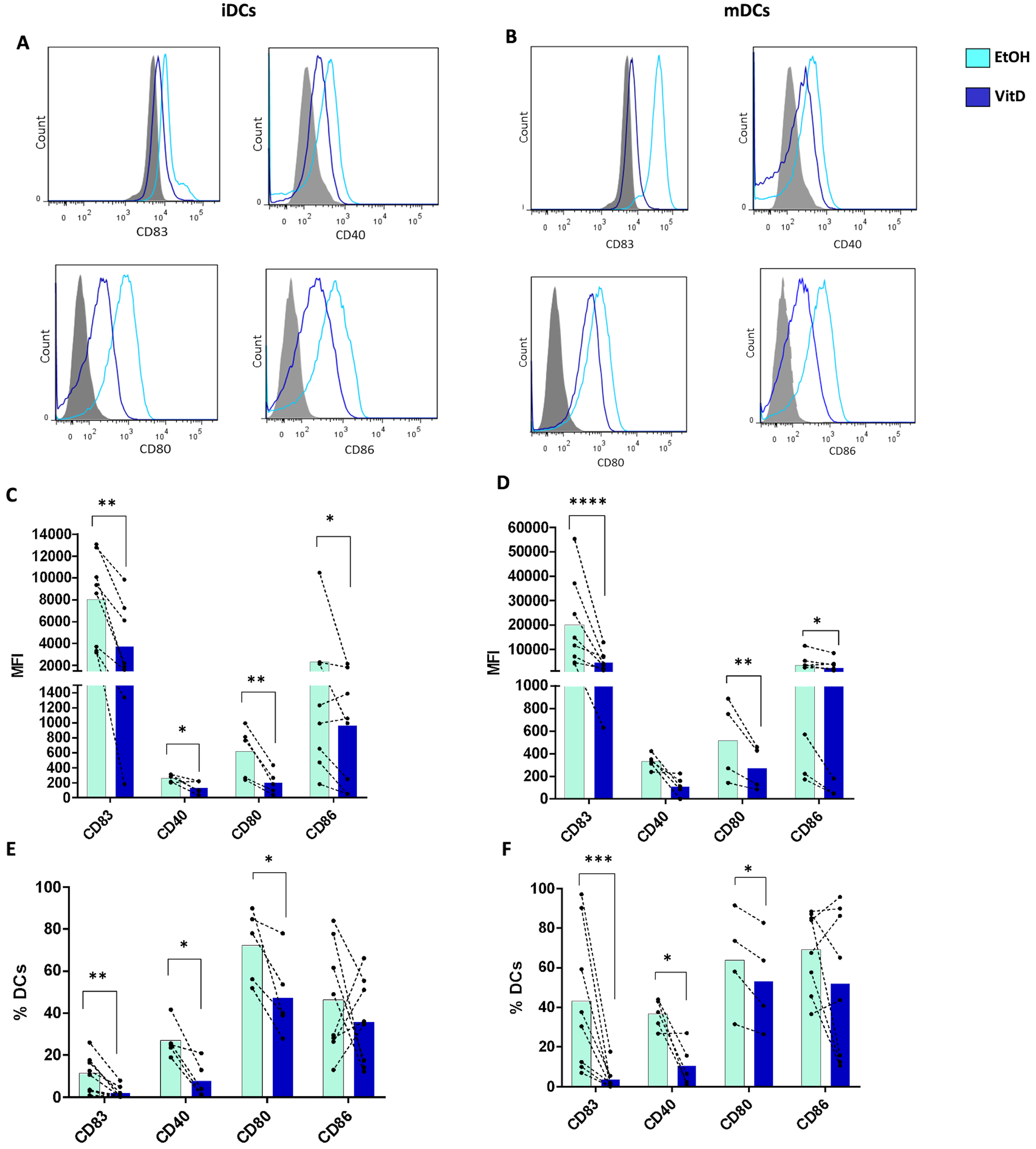

Supplement: S4 Fig — iDCs (left panel) and LmDCs (right panel) were differentiated in the presence of 5×10^−9^ M of calcitriol or 0.01% vol/vol EtOH (control vehicle). (A and B) Representative overlay histograms comparing the expression of CD83, CD40, CD80, and CD86 markers on unstained EtOH treated cells (grey fill), calcitriol treated cells (dark blued lines), and EtOH treated cells (cyan lines) in both iDCs (A) and LmDCs (B). In the bar graph, cyan bars correspond to cells treated with EtOH and blue bars to cells treated with calcitriol. MFI (C and D) and % (E and F) in iDCs and LmDCs, correspond to the expression of different markers in 4 to 8 individuals. Statistical analysis was performed using a Ratio paired test. Bars represent the mean*P < 0.05, **P < 0.01, ***P < 0.001, and ****P < 0.0001. (TIFF) [file pone.0269932.s004.tiff]

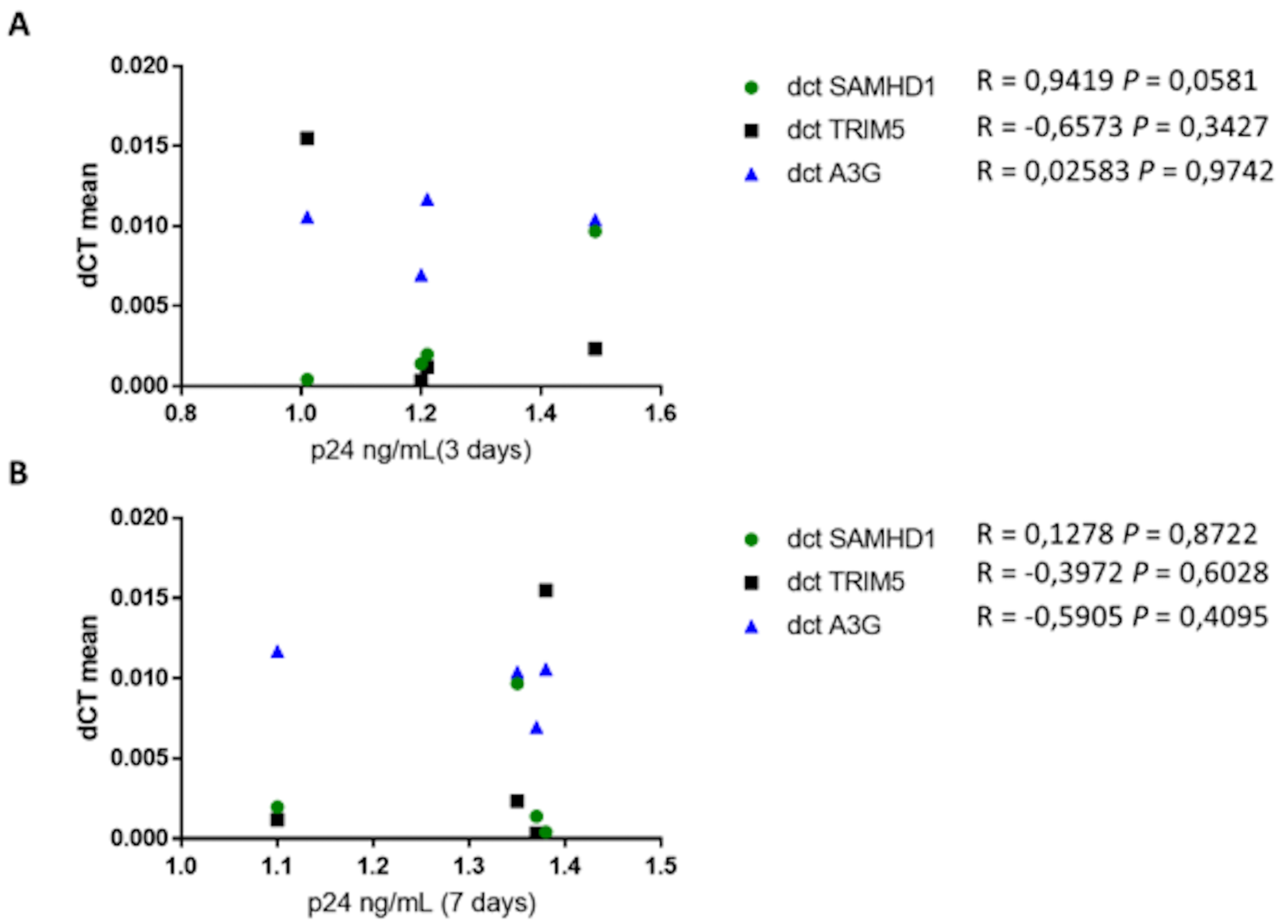

Supplement: S5 Fig — The p24 levels were measured by ELISA test from the supernatant of the infected CD4+ T cells cultures (n = 4) at 3 (A) and 7 days (B) pos-infection. The gene expression levels for SAMHD1 (green circles), TRIM5 (black square), and APOBEC3G (A3G) (blue triangle) were measures by q-PCR using ACTB as a reference gene. Correlations were evaluated using the Pearson coefficient rank (r). (TIFF) [file pone.0269932.s005.tiff]

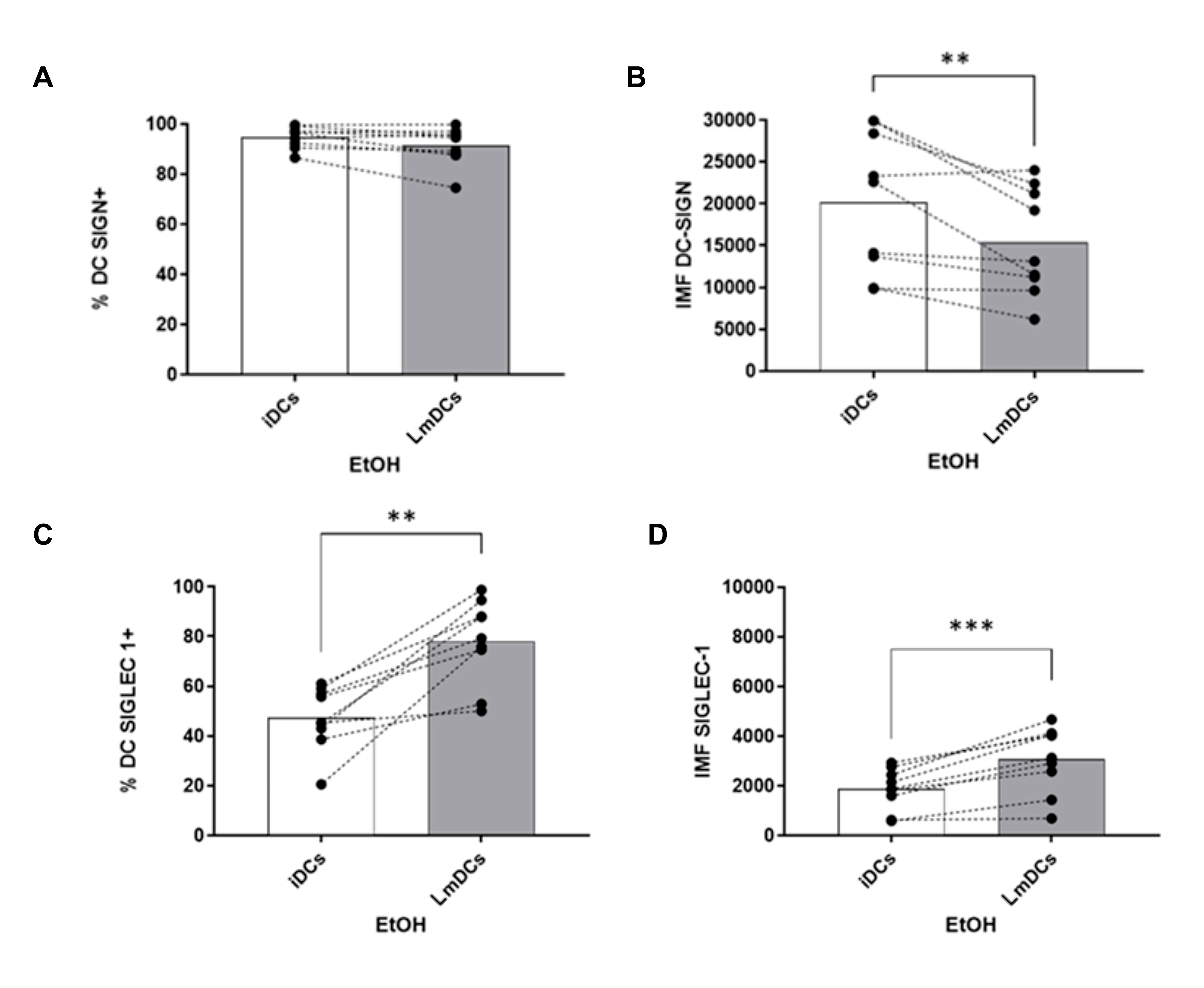

Supplement: S6 Fig — About the DC-SIGN receptor, the MFI was decreased significantly upon maturation (p = 0,0084), while the percentage of cells did not change. For the SIGLEC-1 expression, both percentage and MFI increased upon maturation, (p = 0,0026% and p = 0,0003 MFI). (TIFF) [file pone.0269932.s006.tiff]
